# Supplementary material for: Gardenia jasminoides Extract, with a Melatonin-like Activity, Protects against Digital Stress and Reverses Signs of Aging
Source: Int J Mol Sci. 2023 Mar 3;24(5):4948. doi: 10.3390/ijms24054948 (PMC10003113; doi:10.3390/ijms24054948)
Supplement: Supplementary file 1 [file ijms-24-04948-s001.zip › ijms-2198914-supplementary.pdf]

International Journal of *Molecular Sciences*

MDPI

Supplementary Data

Gardenia Jasminoides Extract, with a Melatonin-Like Activity, Protects against Digital Stress and Reverses Signs of Aging

Quantification of crocin isomers in *Gardenia jasminoides* J. Ellis fruit extract by HPLC/UV

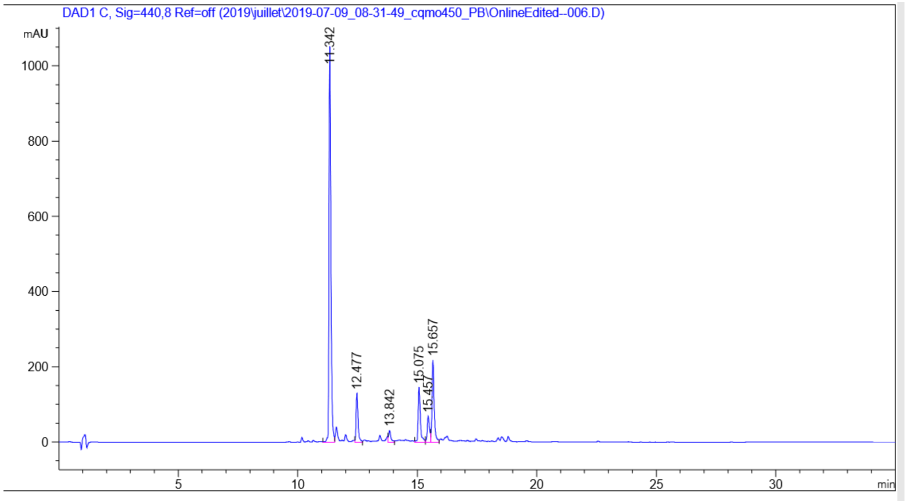

Figure S1: Chromatogram at 440nm of *Gardenia jasminoides* J. Ellis fruit extract, ref 1EAA8869, batch n° A187/022/A18

Table S1. List of crocin derivatives identified and quantified in *Gardenia jasminoides* fruit extract. ref 1EAA8869. batch n° A187/022/A18. Quantification performed in trans-crocetin di-gentiobioside.

Time (min)	Crocin Isomers
11.342	Trans crocetin di-gentiobioside
12.477	Trans crocetin gentiobioside-glucoside
13.842	Crocetin gentiobioside
15.075	Cis crocetin di-gentiobioside
15.457	Crocetin gentiobioside
15.657	Cis crocetin di-gentiobioside-glucoside
17.000	Crocetin glucoside
